# miR-429 Suppresses Endometrial Cancer Progression and Drug Resistance via DDX53

**DOI:** 10.3390/jpm13091302

**Published:** 2023-08-25

**Authors:** Kyung-Jun Lee, Nitya Singh, Michael Bizuneh, Nam-Hyeok Kim, Hyeong Su Kim, Youngmi Kim, Jae-Jun Lee, Jung Han Kim, Jiye Kim, Soo Young Jeong, Hye-Yon Cho, Sung Taek Park

**Affiliations:** 1 Institute of New Frontier Research Team, Hallym University, Chuncheon 24252, Republic of Korea; rudwns0222@naver.com (K.-J.L.); nityasingh75@gmail.com (N.S.); nhkim2429@gmail.com (N.-H.K.); nep2n@hallym.or.kr (H.S.K.); kym8389j@hallym.ac.kr (Y.K.); iloveu59@hallym.or.kr (J.-J.L.); ohora_87@naver.com (S.Y.J.); 2 Division of Hemato-Oncology, Department of Internal Medicine, Kangnam Sacred-Heart Hospital, Hallym University Medical Center, Hallym University College of Medicine, Seoul 07441, Republic of Korea; harricil@hallym.or.kr; 3 Departments of Anesthesiology and Pain Medicine, Chuncheon Sacred-Heart Hospital, Hallym University Medical Center, Hallym University College of Medicine, Chuncheon 24253, Republic of Korea; 4 Department of Obstetrics and Gynecology, Kangnam Sacred-Heart Hospital, Hallym University Medical Center, Hallym University College of Medicine, Seoul 07441, Republic of Korea; sdjiye@hanmail.net; 5 Department of Obstetrics and Gynecology, Dongtan Sacred-Heart Hospital, Hallym University Medical Center, Hallym University College of Medicine, Kyeonggido 18450, Republic of Korea

**Keywords:** endometrial cancer, DDX53, miR-429, anti-cancer drug-resistance

## Abstract

(1) Background: To examine miR-429-meditated DEAD (Asp-Glu-Ala-Asp) box polypeptide 53 (DDX53) function in endometrial cancer (EC). (2) Methods: DDX53 and miR-429 levels were measured using quantitative real-time polymerase chain reaction and western blotting assays, cell invasion and migration using Transwell invasion and wound healing assays, and cell proliferation using colony-forming/proliferation assays. A murine xenograft model was also generated to examine miR-429 and DDX53 functions in vivo. (3) Results: DDX53 overexpression (OE) promoted key cancer phenotypes (proliferation, migration, and invasion) in EC, while in vivo, DDX53 OE hindered tumor growth in the murine xenograft model. Moreover, miR-429 was identified as a novel miRNA-targeting DDX53, which suppressed EC proliferation and invasion. (4) Conclusions: DDX53 and miR-429 regulatory mechanisms could provide novel molecular therapies for EC.

## 1. Introduction

The DEAD (Asp-Glu-Ala-Asp) box helicase family is the largest helicase family in eukaryotes. The DEAD-box RNA helicase protein family functions in almost all aspects of eukaryotic RNA metabolism and thus, takes part in the progression of a lot of diseases, such as virus infection, inflammation, and cancer [1,2]

Belonging to the DEAD-box RNA helicase protein family, DDX53 (DEAD (Asp-Glu-Ala-Asp) box polypeptide 53) is implicated in several cell processes, including RNA metabolism and gene regulation [3]. DDX53, also known as cancer/testis antigen cancer-associated gene (CAGE), is only expressed in normal testes, suggesting it is a possible target in gynecological cancer therapy. While CAGE has been identified in gastric cancer, hematological malignancy, and EC sera [4,5,6], DDX53 associations with EC remain unclear, therefore, its role in EC pathogenesis and progression requires investigation.

MicroRNAs (miRNAs) are small non-coding, gene-regulating molecules [7], which bind to messenger RNA (mRNA) to suppress gene expression [7]. miRNAs are involved in a wide range of cellular biological processes by binding to 3′-UTR (3′-untranslated region) of the target mRNAs, which induces the translational inhibition and/or degradation of mRNAs [8,9,10,11]. The correlation between miRNA expression and cancer has been reported in various cancers. In colon cancer, over-expression of miR-155 contributes to carcinogenesis via down-regulation of DNA mismatch repair protein MLH1 [12]. In non-small cell lung cancer, deficiency of miR-324a was reported to be related to poor survival [13]. Also, epithelial-to-mesenchymal transition (EMT) in non-small cell lung cancer is associated with down-regulation of miR-30a [14].

The miRNA-200 family has been documented to be involved in the tumorigenesis and progression of human cancers [15]. As a member of the miRNA-200 family, there is accumulating research focusing on the roles of miR-429 in tumorigenesis and drug resistance in diverse cancers [16,17,18,19]. miR-429 expression is negatively correlated with cancer progression in colorectal cancer [20]. Overexpression of miR-429 may suppress apoptosis of colorectal cancer cells and the tumor-inhibiting effect of miR-429 may function by targeting Onecut2 [21]. Also, miR429 functions as a tumor suppressor by downregulation of transcriptional repressor ZEB1 in oral squamous cell carcinoma, breast cancer, and osteosarcoma [22,23,24]. In esophageal carcinoma, miR-429 suppresses invasion and promotes apoptosis via targeting Bcl-2 and SP1 [25]. MiR-429 regulates the metastasis and EMT of hepatocellular cancer cells by targeting RAB23 [26]. According to the research, miR-429 may play important roles in multiple tumor diseases by regulating different target genes’ expression.

Endometrial cancer (EC) is one of the most common gynecological cancers, with increasing global incidence rates [27]. Early-stage disease patients have better prognosis outcomes with 5-year survival rates between 80% and 95%. However, those with advanced disease do not respond well to standard treatments and their prognosis outcomes remain low [28]. Therefore, a comprehensive investigation of the molecular mechanisms underpinning EC is required for more effective therapeutic development.

In several cancers, DDX53 is reported to be controlled by miRNAs, which suggests it may be a promising candidate in anti-cancer drug development [29,30]. Cancer/testis antigen DDX53 is found in the sera of various cancers, and the expression of DDX53 is regulated by methylation [4,5,6,31]. DDX53 acts as an oncogene and regulates the expression of cyclins [32]. In addition, DDX53 confers resistance to anti-cancer drugs by regulation of miR feedback loops, such as miR-200b-DDX53 and miR-217-DDX53 [29,30,33]. A miR-200b inhibitor induced the direct regulation of SOX-2 by DDX53, which suggests that DDX53 may serve as an immunotherapeutic target for regulating cancer stem-like properties of melanomas [34]. However, DDX53 and miRNA-DDX53 axis roles in EC are poorly characterized.

Therefore, we examined DDX53 functions in several EC cell lines and explored miR-429 in targeting DDX53. We also characterized the DDX53/miR-429 axis in anti-cancer drug resistance.

## 2. Materials and Methods

### 2.1. Cells and Reagents

EC (HEC-1 and Ishikawa) and carcinosarcoma (SNU-685 and SNU-539) uterine cell lines were supplied by the Korean Cell Line Bank. Ishikawa and HEC-1 cells were grown in Dulbecco’s Modified Eagle Medium and McCoy’s 5A media, respectively (Welgene, Gyeongsan-si, Republic of Korea), while SNU-685 and SNU-539 cells were maintained in RPMI1640 (Welgene). All media contained 10% fetal bovine serum (FBS, Invitrogen, Waltham, MA, USA) plus 1% penicillin. Cells were grown in humidified air with a temperature of 37 °C and with 5% of CO_2_. Transfection reagents (jetPRIME^®^ and Interferin^®^) were supplied by PolyPlus-Transfection (Illkirch, France). Antibodies against E-cadherin (#3195s), Snail (#3879s), DDX53 (#AMAb91489), and actin (#3700s) came from CST (Danvers, MA, USA) and ATLAS Antibodies (Bromma, Sweden). CCK-8 assays were supplied by Medifab (Geumcheon-gu, Republic of Korea), and Sigma-Aldrich (Burlington, MA, USA) supplied the paclitaxel.

### 2.2. Transfections

MiR-429 mimic, inhibitor, and negative control (miR-NC and anti-miR-NC) reagents came from Bioneer (Daejeon, Republic of Korea). Small interfering RNA (siRNA) molecules against DDX53, si-NC, NC vector, and the DDX53 overexpression (OE) plasmid were supplied by VectorBuilder (Chicago, IL, USA). Reagents were transfected into HEC-1 and SNU-539 cells, and cells were collected after 48 h. Transfection rates were evaluated using RFP (red fluorescent protein) visualization for overexpressed cells, and western blot (WB) and Reverse Transcription-quantitative Polymerase Chain Reaction (RT-qPCR) were used for microRNA inhibitors, mimics, and target gene siRNAs. Transfection rates were over 80%. All assays were conducted three times.

### 2.3. Quantitative Real-Time PCR (qRT-PCR)

Using an easy-Blue kit (Invitrogen), RNA was extracted, and first-strand cDNA was generated by reverse transcription (Maxime™ RT PreMix (Random Primer), while Mir-X™ miRNA First-Strand Synthesis reagent (TaKaRa, Kusatsu, Japan) was used for miRNA. An SYBR Green PCR kit was used for RT-PCR on Rotor-Gene Q instrumentation (Qiagen, Hilden, Germany). Actin was used as an internal normalization control, and fold change was calculated using the 2^−ΔΔCt^ strategy. Primers were: DDX53 for; 5′-TGGCCAGATACTGTACGTCAA-3′ and Rev 5′-CTTGGGTGAGAGCTCGTTTTT-3′; Actin For 5′-AAACTGGAACGGTGAAGG-3′ and Rev 5′-TGCAATCAAAGTCCTCGG-3′; and miR-429 For 5′-TAATACTGTCTGGTAAAACCGT-3′ and a universal Rev primer. Universal Rev primers were supplied by Mir-X™ miRNA First-Strand Synthesis reagent (Takara, Kusatsu, Japan). To conduct qRT-PCR, a three-step protocol was followed with the following conditions: denaturation at 94 °C for 15 s, annealing at 55 °C for 30 s, and elongation at 70 °C for 30 s. The process was repeated for a total of 40 cycles. All assays were performed in triplicate. The expression levels were relative to the fold changes of the corresponding controls, which were defined as 1.0

### 2.4. Western Blotting (WB)

A radio-immunoprecipitation assay RIPA buffer (iNtRON, Kyeonggi-do, Republic of Korea) plus an Xpert Protease Inhibitor Cocktail Solution (GenDEPOT, Seoul, Republic of Korea) was used to generate total protein lysates. Protein concentrations were then determined using a Bicinchoninic Acid assay kit (Thermo Scientific™, Waltham, MA, USA); the proteins were then electrophoretically separated by sodium dodecyl sulfate-polyacrylamide gel electrophoresis and transferred to polyvinylidene difluoride membranes (Bio-Rad, Hercules, CA, USA). Membranes, blocked in 5% skim milk for 1 h, were incubated at 4 °C overnight with primary antibodies against DDX53 (1:500), Snail (1:2000), Multi-Drug Resistance 1 (MDR1, 1:500), E-cadherin (1:5000), and actin (1:10,000). Membranes were incubated with secondary horseradish peroxidase (HRP)-linked secondary antibodies (goat anti-mouse IgG F(ab’) 2 pAb (HRP conjugate) (Enzo, Farmingdale, NY, USA, 1:3000), and goat anti-rabbit IgG, pAb (HRP conjugate) (Enzo, Farmingdale, NY, USA, 1:3000)) in 5% skim milk at room temperature for 2 h. After the incubation, membranes were washed 3 times (10 min each), and signals were detected by a western Pico-enhanced chemiluminescent kit (LPS Solution, Burlington, MA, USA), followed by ImageQuant LAS 500 (Cytiva, Marlborough, MA, USA) processing. Image J was used to evaluate band density.

### 2.5. Luciferase Reporter Assays

Potential miR-429 binding/mutant sites in the 3′-untranslated region (UTR) of DDX53 were generated in luciferase reporter plasmids (pDDX53-WT or pDDX53-MUT) (Vectorbuilder). Assays were performed in 96-well plates using a Luciferase Reporter Assay kit (Promega, Madison, WI, USA) according to the manufacturer’s guidance. pDDX53-WT or pDDX53-MUT plasmids were co-transfected (50 nM Interferin^®^) with miR-429 inhibitor, miR-429 mimic, and miR-NC reagents. All plasmid and reagents were transfected at a final concentration of 50 nM, using INTERFERin^®^ (PolyPlus, Illkirch, France). After 2 days, a GloMax^®^ Discover Microplate Reader (Promega) was used to record absorbance data. The data were relative to the fold change of the corresponding control groups defined as 1.0.

### 2.6. Cell Viability

We determined relative cell viability using tetrazolium monosodium salt (WST-8 Viability Assay Kit, Medifab, Seoul, Republic of Korea) and by incubating 1500–2000 cells in 96-well plates for 1 day and adding CCK-8 solution (10:1 medium-to-reagent ratio). Absorbance at 450 nm was measured after 2 h. The assays were performed three times to ensure accuracy.

### 2.7. Drug Treatment Assays

Approximately 3000–5000 cells/well grown in 96-well plates for 1 day were treated with 10^−4^ to 10 μM paclitaxel (diluted in culture medium plus 2% FBS). Paclitaxel (PTX), sold under the brand name Taxol among others, is a chemotherapy medication used to treat ovarian cancer, and other cancer. After 2 days, 10 μL WST-8 solution was added and plates were incubated for 120 min. Relative cell viability was recorded at 450 nm and data were normalized to control cells without paclitaxel.

### 2.8. Scratch Wound Healing Assays

EC cell migration was examined using wound healing assays. Approximately 5 × 10^5^ transfected or transduced cells were grown in 6-well plates until 100% confluent, wounded by scraping monolayers, then washed 3 times in FBS-free medium, and cultured in a complete medium. Wound areas were recorded at 0 h and 48 h; migration was estimated by subtracting wound areas at 48 h from 0 h wound areas. Wound areas were measured using Image J. Three independent assays were assayed.

### 2.9. Invasion Assays

Cell invasion was evaluated using 8-μm pore Transwell chambers (SPL, Pocheon-si, Republic of Korea) and matrigel (Corning, Glendale, AZ, USA). At 2 days post-transfection/transduction, cells (serum-free media) were added to Transwell chambers plus matrigel (2–10 μg/mL), while complete medium (plus 10% FBS) was added to lower chambers. After 2 days, migrated/invaded cells were fixed in 4% paraformaldehyde, stained in 0.1% crystal violet (Sigma-Aldrich, Burlington, MA, USA), and enumerated by microscopy. Numbers were averaged across three independent assays.

### 2.10. Colony Formation Assays

For colony formation, cells (10^3^/well) grown in 6-well plates had their media refreshed every 3 days for 10 days. Colonies, fixed in 4% paraformaldehyde for 10 min, were stained in 0.1% crystal violet (Crystal violet, Sigma-Aldrich, Burlington, MA, USA).

### 2.11. In Vivo Studies

In this experiment, we evaluated the effect of DDX53 overexpression or downregulation on primary tumor growth. Our Institutional Animal Care and Use Committee (Hallym University (2022-38) sanctioned the study and provided guidelines. Four-week-old female BALB/C, specific-pathogen-free nude mice were provided by DooYeol Biotech (Seoul, Republic of Korea). To generate the model, HEC-1 cells (in 50 μL phosphate buffered saline) were added to 50% matrigel (Corning^®^ Matrigel^®^ Matrix, Corning, NY, USA) and subcutaneously injected at 4 × 10^6^ cells/site into the hind flanks of mice. When average tumor volumes were approximately 80 mm^3^, animals were randomly assigned to control and experimental groups. The latter group received intratumoral injections of 10 μg miR-429 inhibitor, DDX53 siRNA, or DDX53 OE plasmid (Bioneer) using an in vivo-jetPEI^®^ system (PolyPlus), with 5 injections every 2 days for 11 days. Every day, tumor volumes were measured by digital caliper, and volumes were calculated (length × width^2^ × 0.5 (mm)). After 11 days, snap-frozen tumor samples were stored at −80 °C. To ensure optimal conditions for the mice, the rooms were kept at a controlled temperature (20–24 °C) and humidity range (45–65%) with a 12-h light-dark cycle. A total of 64 mice were used, consisting of 13 mock, 5 DDX53 O/E, 10 miR-NC, 10 miR-429 inhibitor, 12 miR-429 inhibitors + control siRNA, and 14 miR-429 inhibitors + DDX53 siRNA.

### 2.12. Statistics

Experiments/assays were performed in triplicate and data were represented as the mean ± standard error of the mean. Prism (Ver. 8.0) was used for analyses. Student’s t- and one-way analysis of variance-tests were used for two- and multiple-group comparisons, respectively. Two-sided tests were performed, and significance was accepted at *p* < 0.05. In all graphs and figures, error bars represent the mean ± standard error of the mean from at least 3 independent experiments/assays. NS = non-significant * *p* ≤ 0.05, ** *p* ≤ 0.01, *** *p* ≤ 0.001, and **** *p* ≤ 0.0001.

## 3. Results

### 3.1. DDX53 Positively Correlates with EC Progression and Metastasis

**Figure 1:**
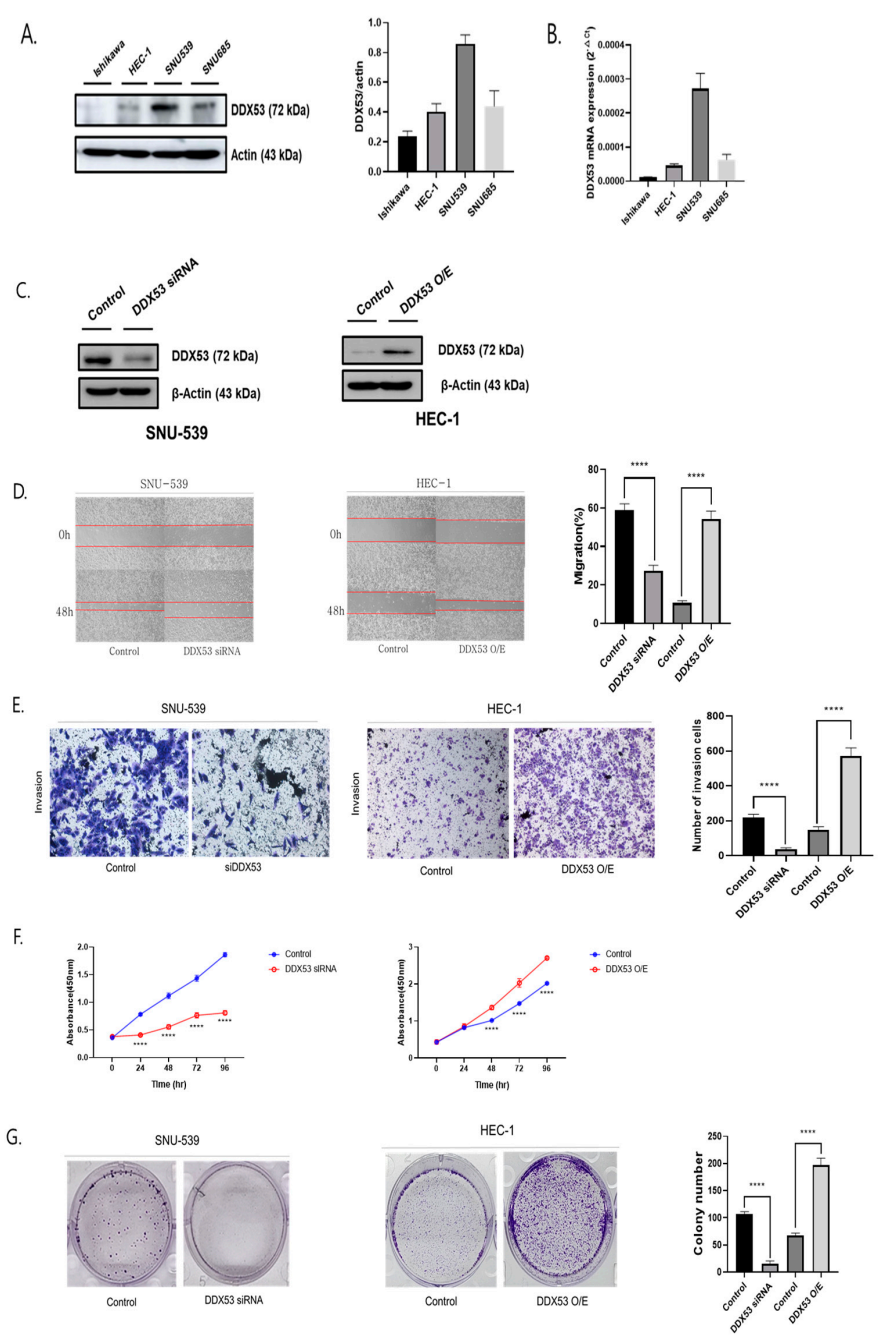
To analyze the two groups (**B**,**D**,**E**,**G**), we utilized the unpaired *t*-test. For the three-group analysis (**F**), we employed the ANOVA *t*-test. These tests yielded the following results: DDX53 regulation contributes to EC cell metastasis and epithelial-mesenchymal transition. (**A**,**B**) Levels of DDX53 in Ishikawa, HEC1, SNU-539, and SNU-685 cells. (**C**) Efficiency of DDX53 overexpression and DDX53 siRNA transfection confirmed using Western Blotting (WB). (**D**) DDX53’s influence on EC migration observed through wound healing assay. The right graph represents the percentage of gaps filled at 48 h compared to 0 h. (**E**) DDX53’s effect on EC invasion assessed using an Invasion assay. The right graph shows the count of invaded cells. (**F**) DDX53 effects on EC cell proliferation. (**G**) DDX53’s impact on EC cell proliferation evaluated through a colony assay. **** *p* ≤ 0.0001.

DDX53 was poorly expressed in HEC-1, Ishikawa, and SNU-685 cells but elevated in SNU-539 cells (Figure 1A,B). To characterize the functions in EC, DDX53 OE and knockdown (KD) studies were performed in HEC-1 and SNU-539 cells, respectively, using WB (Figure 1C). Based on our experimental result, DDX53 was the most highly expressed in SNU-539 and the least expressed in HEC-1 and Ishikawa. Under the down-regulation of DDX53, we could not detect an EMT marker (e.g., snail) in Ishikawa. So, we only used HEC-1 and SNU-539 in the final experiments. DDX53 OE in HEC-1 cells increased cell migration and invasion rates, while DDX53 KD in SNU-539 cells decreased rates (Figure 1D,E). Additionally, DDX53 OE and KD increased and decreased cell proliferation, respectively (Figure 1F,G). Tumors generated by HEC-1 cells transfected with the DDX53 OE plasmid were bigger when compared with controls. Thus, DDX53 putatively regulated EC progression and metastasis via epithelial-mesenchymal transition (EMT), where cancer cells lost epithelial polarity and transitioned to a mesenchymal phenotype.

### 3.2. DDX53 Upregulates Chemoresistance and Mesenchymal Markers in EC Cells

**Figure 2:**
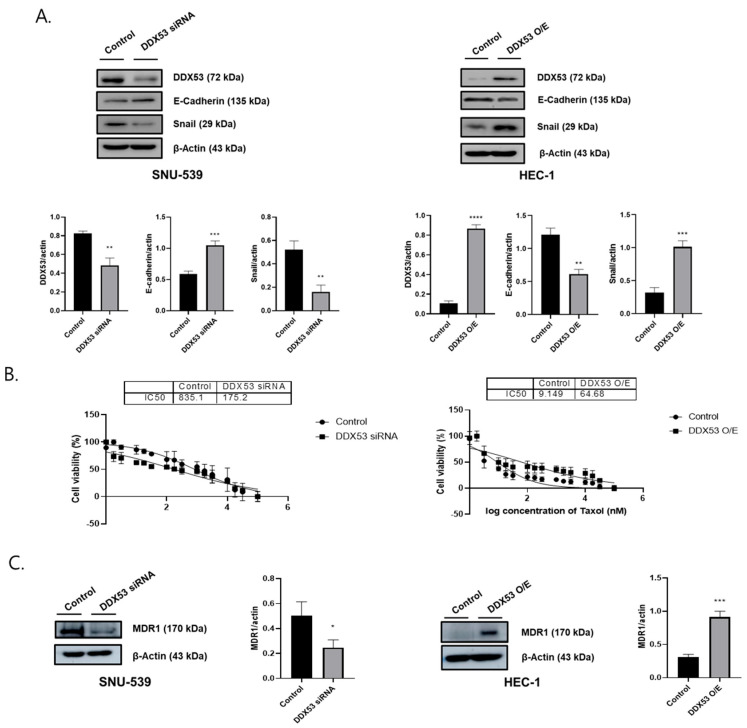
DDX53 regulates epithelial-mesenchymal transition and chemoresistance. (**A**) DDX53’s influence on EMT in EC cells, confirmed via WB. (**B**) DDX53’s impact on EC chemoresistance, demonstrated by IC50 values using GraphPad. (**C**) DDX53’s effects on MDR1 levels in EC cells, as validated through WB. All experiments were conducted with triplicate repetitions. An unpaired *t*-test was used for the comparison of the two groups. * *p* ≤ 0.05, ** *p* ≤ 0.01, *** *p* ≤ 0.001, and **** *p* ≤ 0.0001.

From our data, DDX53 appeared to modulate EMT and chemoresistance in EC cells; therefore, we confirmed DDX53 functions during EMT in EC cells using WB. In HEC-1, cells with DDX53 overexpression, reduced E-cadherin (epithelial marker), and increased Snail (mesenchymal marker) levels were observed (Figure 2A). However, when using the DDX53 KD plasmid in SNU-539 cells, the results were the opposite.

To examine DDX53’s effects on chemoresistance, the anti-cancer agent paclitaxel was used (10^−1^ to 10^5^ nM concentration range). As shown in Figure 2B, DDX53 OE increased paclitaxel IC50 values (9.149 vs. 64.68 nM), while DDX53 KD decreased values (835.1 vs. 175.2 nM). DDX53 OE increased MDR1 levels, while DDX53 KD caused the opposite (Figure 2C). Thus, DDX53 putatively regulated EMT and chemoresistance in EC cells by modulating MDR1.

**Figure 3:**
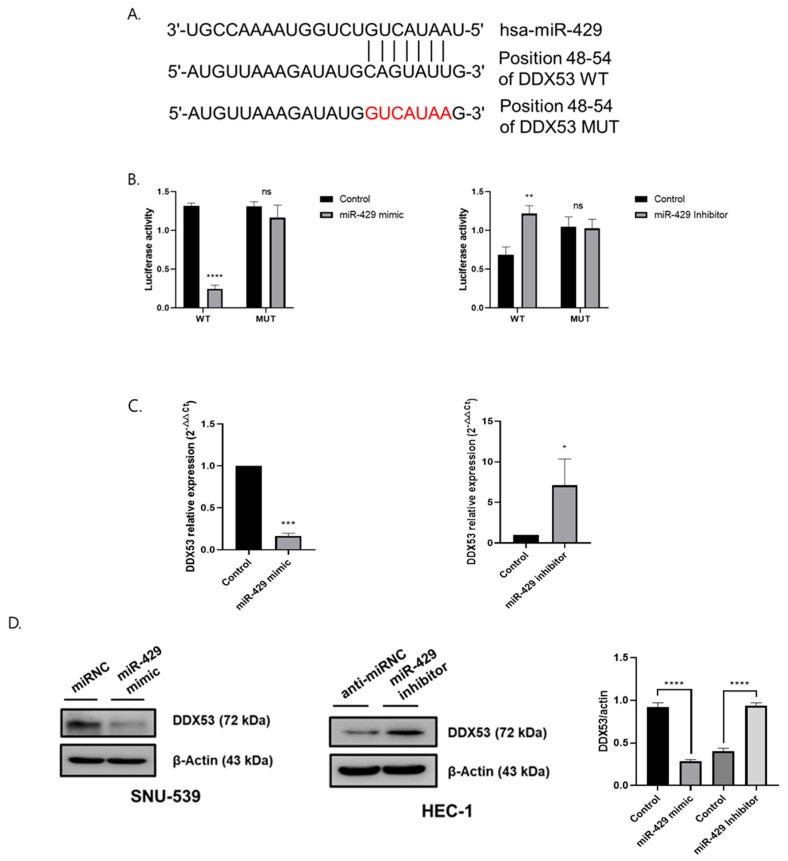
DDX53 is targeted by MiR-429. (**A**) Binding between miR-429 and DDX53. (**B**) The miR-429 mimic and inhibitor regulate DDX53 luciferase reporter activity. (**C**,**D**) MiR-429 regulates DDX53, confirmed by WB for protein levels RT-qPCR for mRNA levels in EC cells. All experiments were conducted with triplicate repetitions. An unpaired *t*-test was used for the comparison of the two groups. ns = non-significant, * *p* ≤ 0.05, ** *p* ≤ 0.01, *** *p* ≤ 0.001, and **** *p* ≤ 0.0001.

From our results, miR-429 negatively regulated DDX53 via its 3′-UTR site (Figure 3A). Two putative miR-429 binding sites (at 48–53 residues) in the DDX53 3′-UTR were identified using TargetScan and miRDB databases. Then, we used luciferase reporter assays to confirm in vitro interactions between miR-429 and the DDX53 3′-UTR in HEC-1 and SNU-539 cells (Figure 3B). MiR-429 bound to the DDX53 3′-UTR; luciferase activity of the WT DDX53 3′-UTR luciferase reporter was reduced and increased by the miR-429 mimic and inhibitor, respectively, but mutant DDX53 3′-UTR luciferase reporter activity was unchanged. Furthermore, DDX53 mRNA and protein levels were significantly reduced by miR-429 OE, while miR-429 KD increased these levels (Figure 3C,D). Therefore, DDX53 was putatively targeted by miR-429.

### 3.3. MiR-429 Regulates EC Metastasis and Proliferation by Targeting DDX53

**Figure 4:**
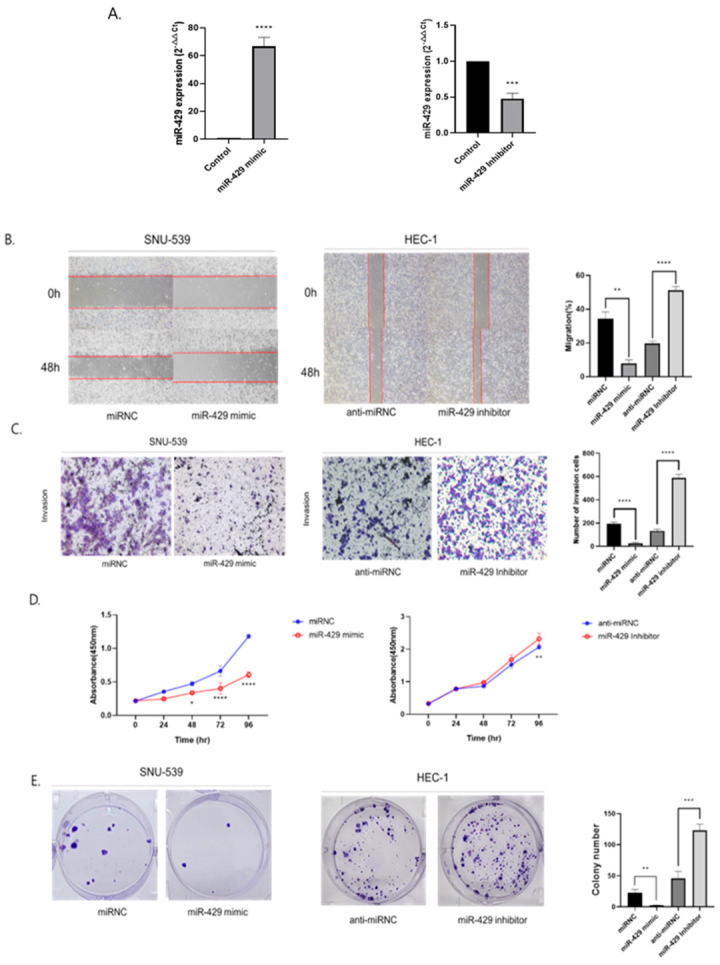
MiR-429 regulates EMT in EC cells. (**A**) Good transfection efficiency of the miR-429 mimic and miR-429 inhibitor (**B**) Wound healing assays were employed to gauge EC migration, while the corresponding right graph represents gap closure percentages from 0 h to 48 h. (**C**) Invasion assays were conducted to analyze EC invasion, and the corresponding right graph enumerates the count of invaded cells. (**D**) DDX53′s effects on EC cell proliferation. (**E**) Colony assays were employed to assess EC cell proliferation, with the corresponding right graph quantifying the number of formed colonies. To perform the two-group analysis (**A**,**B**,**C**,**E**), the unpaired *t*-test was utilized. Meanwhile, for the three-group analysis (**D**), the ANOVA *t*-test was employed. * *p* ≤ 0.05, ** *p* ≤ 0.01, *** *p* ≤ 0.001, and **** *p* ≤ 0.0001.

After we confirmed binding between DDX53 and miR-429 in EC cells, we examined miR-429 functions on EMT and metastasis in SNU-539 and HEC-1 cells transfected with the miR-429 mimic and inhibitor (miRNC or anti-miRNC, respectively). After 2 days, good transfection efficiency was observed (Figure 4A). We then examined miR-429 effects on EC migration/invasion and observed (Figure 4B,C) that forced miR-429 expression significantly inhibited EC metastasis. Next, we evaluated miR-429 effects on EC proliferation and showed (Figure 4D,E) that miR-429 OE significantly weakened EC cell proliferation, while miR-429 KD enhanced proliferation. Thus, miR-429 was potentially implicated in EC cell proliferation, metastasis, and EMT.

### 3.4. Downregulated miR-429 Contributes to EMT and Increased Anti-Cancer Sensitivity in EC Cells

**Figure 5:**
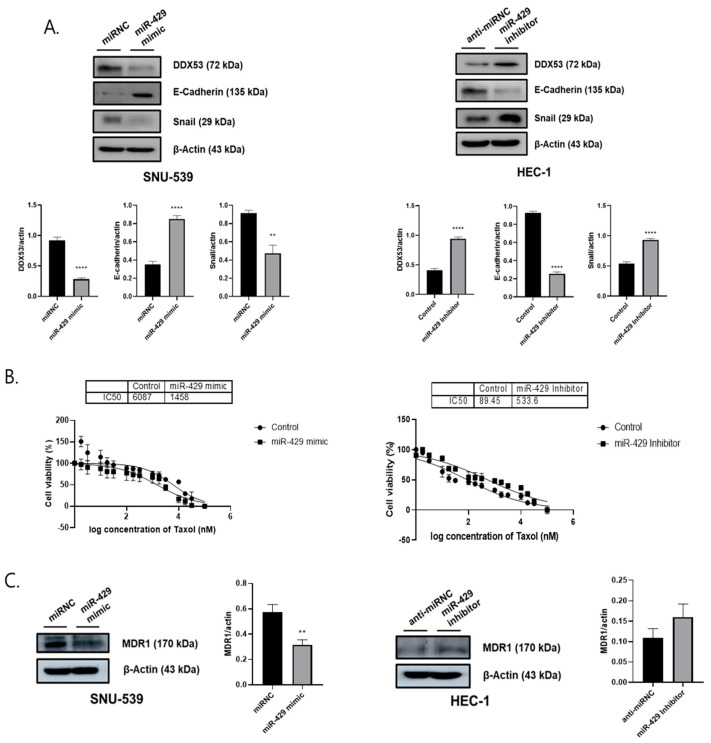
MiR-429 suppresses mesenchymal markers and chemoresistance. (**A**) MiR-429’s effects on epithelial-mesenchymal transition in EC cells. Verified using WB. (**B**) MiR-429’s effects on anti-drug capacity in EC cells. IC50 values were calculated employing GraphPad. (**C**) MDR1 level effects via miR-429 regulation. Verified using WB. To conduct a two-group analysis, we utilized an unpaired *t*-test. ** *p* ≤ 0.01, and **** *p* ≤ 0.0001.

From our results, miR-429 enhanced epithelial marker levels and suppressed chemoresistance in EC cells. WB assays examining E-cadherin and Snail levels in EC cells expressing the miR-429 mimic or inhibitor showed that miR-429 upregulation in SNU-539 cells induces transformation of MET, the reverse of EMT, while miR-429 downregulation facilitated EMT in HEC-1 cells (Figure 5A). MiR-429 effects toward chemoresistance were then examined using paclitaxel (10^−1^–10^5^ nM) and showed (Figure 5B) that upregulated miR-429 decreased paclitaxel IC50 values (6087 nM vs. 1458 nM), whereas downregulated miR-429 elevated values (89.45 nM vs. 533.6 nM) (Figure 5B). Our WB analyses indicated that miR-429 downregulated MDR1 levels (Figure 5C). Thus, miR-429 functions in EC may be facilitated via DDX53 targeting.

### 3.5. The miR-429/DDX53 Axis Regulates In Vivo EC Tumor Progression

**Figure 6:**
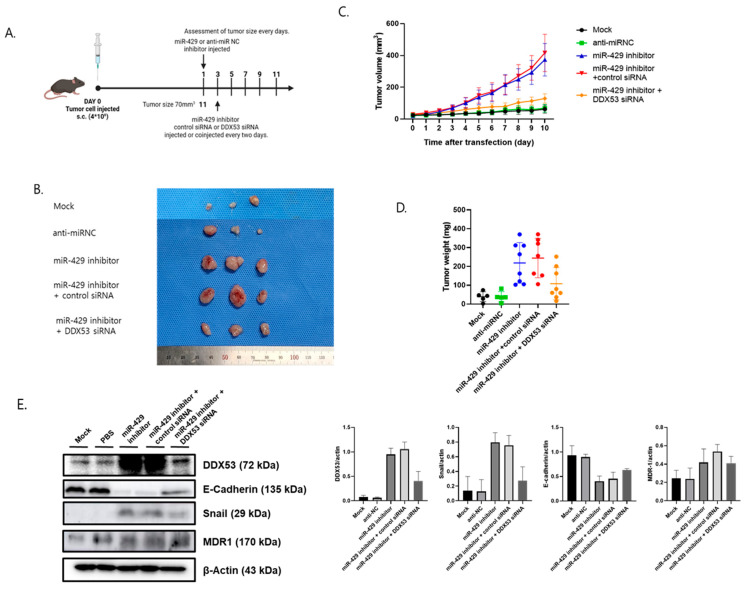
In vivo miR-429/DDX53 axis therapeutic effects. (**A**) Transfection schedule. (**B**) Image showing tumors in nude mice injected with HEC-1 cell. (**C**) Tumor volume alterations across groups during miR-429 inhibitor and DDX53 siRNA transfections. (**D**) Tumor weight alterations across groups. (**E**) Protein levels across groups. To analyze the two groups (**D**,**E**), we utilized the unpaired *t*-test. For the three-group analysis (**C**), we employed the ANOVA *t*-test.

We examined miR-429/DDX53 axis effects on in vivo tumorigenesis in a xenograft tumor model. We first administered miR-429 inhibitors or anti-miR inhibitor NC and then administered control siRNA or DDX53 RNA 48 h later (Figure 6A). The mock group, which had formed tumors and received no injections, was the control group. In model animals, tumor size increased when miR-429 was downregulated. Additionally, tumor shrinkage was significantly reduced when DDX53 and miR-429 were simultaneously downregulated (Figure 6B–D). Also, suppressed miR-429 induced reduced E-cadherin and increased Snail levels (Figure 6E). However, these changes were reversed when DDX53 levels were further reduced (Figure 6E). Therefore, the miR-429/DDX53 axis may control EC tumorigenesis in our xenograft model.

## 4. Discussion

From our analyses, DDX53 exhibited variable levels across different EC cell lines. Although DDX53 expression is restricted to normal testicular germline cells, upregulated DDX53 levels were reported in several cancer cell lines [4,5,6,35,36]. DDX53, as a cancer-testis antigen, was also reported to regulate cyclin levels and function as an oncogene [6]. DDX53 was also shown to control anti-cancer drug resistance with miRNAs (miR-200b and miR-217) via negative feedback loops [29,30,34].

To examine DDX53 functions in EC cells, we conducted DDX53 OE and KD studies in HEC-1 and SNU-539 cells, respectively, and showed that DDX53 OE in HEC-1 cells increased key cancer phenotypes, whereas DDX53 KD in SNU-539 cells decreased them. Moreover, in vivo data showed that HEC-1 cells transfected with a DDX53 OE plasmid generated significantly larger tumors when compared with controls. Thus, DDX53 may regulate EC progression and metastasis by inducing epithelial polarity loss and mesenchymal phenotype acquisition, or EMT, in cancer cells [37].

Anti-cancer drug resistance mechanisms mediated by upregulated CAGE have been previously reported [29,30,33]. In taxol or celastrol-resistant cancer cell lines, miR-200b and CAGE generated a regulatory feedback loop, which modulated microtubule-targeting drug responses, and invasive, tumorigenic, and angiogenic potential [30]. In a study on human hepatic cancer cells (SNU-387) and drug-resistant human melanoma Malme3M (Malme3M(R)), chromatin immunoprecipitation demonstrated that CAGE, via histone deacetylase 2 interactions, negatively affected p53 levels in Malme3M(R) cells. Thus, CAGE potentially conferred drug resistance by regulating p53 levels via histone deacetylase 2 [33]. To date, only a limited number of studies have explored the relationship between CAGE and endometrial cancer. A research study conducted using cDNA expression cloning with patients’ sera (SEREX) has revealed that CAGE is expressed in endometrial cancer (seven out of ten cases) and atypical endometrial hyperplasia (one out of three cases) [5]. Moreover, the study found that anti-CAGE antigen was highly positive in microsatellite instability (MSI)-positive endometrial cancer, indicating that CAGE could serve as a useful marker for early diagnosis or prognosis of endometrial cancer with MSI-positivity [5]. Similarly, in vitro, DDX53 was elevated in taxol-resistant cervical cancer cells when compared with HeLa cells (parental cell line); DDX53 upregulated MDR1 levels and promoted anti-cancer drug resistance in cervical cancer [36]. We observed that DDX53 OE caused paclitaxel chemo-resistance via upregulated MDR1 levels in EC cells, thus aberrant DDX53 levels affected EC cell responses to paclitaxel, indicating potential roles in drug resistance.

In in vivo studies, DDX53 OE hindered tumor growth. Moreover, miR-429 suppressed EC proliferation and invasion. MiRNAs may regulate over 30% of gene expression and be responsible for aberrant gene expression in cancer cells [5]. Previously, miRNAs, including miR-200b, miR-217, and miR-335, were reported to regulate DDX53 levels in different cancer cells [5,38]. From our bioinformatics analyses, miR-429 was identified with a strong binding affinity to the DDX53 3′-UTR. Typically, miRNAs base-pair to the target 3′-UTR to either hinder or enhance mRNA translation or degradation, respectively [39]. In our investigation, we confirmed regulatory relationships between miR-429 and DDX53 using luciferase reporter assays. MiR-429 bound to the DDX53 3′-UTR; luciferase activity of the WT DDX53 reporter was reduced and increased by the miR-429 mimic and inhibitor, respectively, but mutant reporter activity was unchanged. Furthermore, DDX53 mRNA and protein levels were significantly reduced by miR-429 OE, while miR-429 KD increased these levels. Therefore, we hypothesized that DDX53 may be controlled by miR-429. However, we only evaluated the effect of DDX53/miR-429 on tumor growth. To clarify the effect of the DDX53/miR-429 axis on tumor metastasis, further evaluation will be required.

MiR-429 is part of the miR-200 family, with some members implicated in EMT in different tumor types [40]. Additionally, negative correlations between miR-429 levels and different cell lines have been reported [23,41,42,43,44], with miR-429 identified as a negative drug resistance indicator in these cancer types. However, miR-429 mechanisms in EC metastasis and chemo-resistance remain unclear. From our results, downregulated miR-429 appeared to promote EC metastasis and chemo-resistance, consistent with previous reports on other cancers, including breast cancer, hepatocellular carcinoma, and osteosarcoma [23,24,26]. We also confirmed DDX53’s tumorigenesis capacity in EC tumors. Our correlation analyses revealed that DDX53 levels were negatively correlated with miR-429 levels, which suggested that DDX53 downregulation by miR-429 significantly decreased EC cell progression and metastasis.

Our report is the first to investigate relationships between miR-429 and DDX53 in EC. DDX53 OE promoted key EC cell phenotypes. We also confirmed that miR-429 suppressed EC growth and metastasis via DDX53 targeting. More importantly, we highlighted the miR-429 and DDX53 negative loop, which contributed to EMT and anti-cancer sensitivity in EC cells. Our findings suggest the need for additional studies examining the link between the DDX53/miR-429 axis and distant metastasis and survival in endometrial cancer.

## 5. Conclusions

The DDX53/miR-429 axis may function as a promising EC molecular target. Further investigations are warranted to highlight axis functions in EC to provide new targeted therapies.

## Data Availability

Not applicable.
